# Efficient solution of particle shape functions for the analysis of powder total scattering data

**DOI:** 10.1107/S1600576722001261

**Published:** 2022-03-18

**Authors:** Alberto Leonardi, Reinhard Neder, Michael Engel

**Affiliations:** a Institute for Multiscale Simulation, IZNF, Friedrich-Alexander-Universität Erlangen-Nürnberg, Cauerstrasse 3, Erlangen, Bavaria 91058, Germany; b Institut für Physik der Kondensierten Materie, Friedrich-Alexander-Universität Erlangen-Nürnberg, Staudstrasse 3, Erlangen, Bavaria 91058, Germany

**Keywords:** shape functions, small-angle scattering, total scattering, pair distribution functions, common volume functions

## Abstract

Structural characterization of powder samples via total scattering methods, in either real or reciprocal space, must take into account the effect of particle shape. Here, the shape contribution of a set of ideally isolated particles to the small-angle scattering (SAS) component of the intensity profile is modelled using the shape function [Svergun & Koch (2003). *Rep. Prog. Phys.* **66**, 1735–1782]. The shape function is obtained by orientational averaging of common volume functions (CVFs) for a discrete set of directions. The effects of particle size and size dispersity are accounted for via scaling of the CVFs and their convolution with the underlying probability distribution. The method is applied to shapes with CVFs expressed analytically or by using discrete tables. The accurate calculation of SAS particle shape contributions up to large momentum transfer demonstrates the reliability and flexibility of modelling shape functions from sets of CVFs. The algorithm presented here is computationally efficient and can be directly incorporated into existing routines for analysis of powder total scattering data.

## Abstract 2

Particle shape functions are necessary to model powder scattering data that are in the form of intensity profiles and pair distribution functions. Here, an efficient method is presented to compute particle shape functions by orientational averaging of common volume functions. Contributions from particle size and size dispersity are accounted for via scaling and convolution.

## 1. Introduction

Accurate structural information is the foundation for the development of materials for heterogeneous catalysis, semi- and superconduction, sensing, and photonic and plasmonic applications (Chen *et al.*, 2017 ▸; Luo & Guo, 2017 ▸; Billinge & Egami, 1993 ▸; Gong *et al.*, 2015 ▸; Hajfathalian *et al.*, 2016 ▸; Gamler *et al.*, 2020 ▸; Leonardi & Engel, 2018 ▸). The synthesis of nanomaterials with fine microstructural features requires advances in powder scattering techniques to fully resolve the particle structure, microstructure and lattice distortion (Scardi *et al.*, 2015 ▸; Solla-Gullon *et al.*, 2015 ▸). Such an analysis is complicated by broadening of the small-angle scattering (SAS) component of the intensity profile due to the small size of nanoparticles, which can no longer be neglected in the analysis of powder scattering data (Scardi *et al.*, 2011 ▸; Li *et al.*, 2016 ▸). In particular, the shape contribution to the SAS component from particles in a powder, which are assumed to be ideally isolated, markedly affects the diffuse scattering between the Bragg peaks as well as the trend of the pair distribution function (PDF) (Olds *et al.*, 2015 ▸). The Fourier transform (FT) of the SAS particle shape contribution, known as the shape function 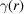 , is used for the analysis of the PDF data to account for the missing scattering information at small momentum transfer in experimental data.

The SAS particle shape contribution is commonly calculated as the orientational average of the form factor, which is the FT of the particle volume. Because orientational averaging can be solved in closed form only for a small number of shapes (Renaud *et al.*, 2009 ▸; Bartlett & Ottewill, 1992 ▸; Svergun & Koch, 2003 ▸), numerical integration must be used to calculate the SAS particle shape contribution for other shapes (Li *et al.*, 2011 ▸, 2016 ▸; Senesi & Lee, 2015*a* ▸). Senesi & Lee (2015*b* ▸) showed that the form factor of an arbitrary polyhedron can be calculated as the sum of the form factors of simple shapes, such as tetrahedra and cubes. More recently, semi-analytic expressions for the form factors of polyhedra were derived by Wuttke (2021 ▸, 2017 ▸), and implemented in the grazing-incidence SAS simulation package *BornAgain* (Pospelov *et al.*, 2020 ▸). Despite these advances, the solution of the shape function via FT of the SAS particle shape contribution remains generally not possible in closed form and is affected by the applied numerical approximations (Svergun & Koch, 2003 ▸). In particular, approximations at large scattering momentum transfer result in significant errors at short distances in the shape function. The SAS shape contribution can be numerically solved only for a finite range of momentum transfer. This introduces systematic errors in the shape function. Furthermore, the degree of discretization applied during numerical integration that is sufficient for an accurate SAS shape contribution within this range is generally not sufficient for an equally accurate shape function.

The shape function describes the probability of two points separated by a distance *r* lying within the particle. It can be calculated as the autocorrelation of the particle shape. Closed-form expressions are available only for a few cases (Guinier, 1956 ▸; Azaroff, 1968 ▸; Svergun & Koch, 2003 ▸; Goodisman, 1980 ▸). A notable example is the shape function of a sphere of diameter *D*, 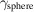 , which has the simple and well known form (Stokes & Wilson, 1942 ▸; Guinier & Fournet, 1955 ▸; Kodama *et al.*, 2006 ▸; Howell *et al.*, 2006 ▸)

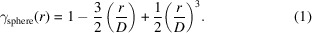

The sphere is a reasonable approximation when the particle shape is unknown, when features of interest in the PDF profile are only weakly affected by the average trend (Polking *et al.*, 2012 ▸; Wang *et al.*, 2013 ▸; Hua *et al.*, 2015 ▸) or when the particle is significantly larger than the longest pair distance accessed via PDF methods (see Section 3.5 for more details). The sphere approximation is also employed by software applications that exploit the small-box modelling method, which approximates the PDF of a nanoparticle by the convolution of the shape function with the PDF of a bulk structure (Farrow *et al.*, 2007 ▸). However, this approximation affects accuracy and precludes PDF methods from capturing microstructural features sensitive to particle shape effects.

Common approaches to evaluate the shape function are based on the trend of the radial distribution function (RDF) (Olds *et al.*, 2015 ▸; Leonardi, 2021 ▸; Egami & Billinge, 2003 ▸). The shape function can be approximated by a polynomial that is optimized in PDF analysis software (Neder & Korsunskiy, 2005 ▸; Korsunskiy & Neder, 2005 ▸; Korsunskiy *et al.*, 2007 ▸; Page *et al.*, 2011 ▸). A more reliable approach is the exploitation of the duality between real-space and reciprocal-space representations of scattering data (Neder & Proffen, 2020 ▸). The intensity profile from the scattering of a powder is modelled by solving the Debye scattering equation (DSE). The shape function is then calculated as the FT of the SAS particle shape contribution (Li *et al.*, 2011 ▸). However, the need for the solution of the DSE makes this approach inefficient. The RDF must be computed with high accuracy during the solution of the DSE for the whole particle even though the reduced experimental data are usually restricted to short pair distances, *e.g.* up to ∼4 nm (Hall & Monot, 1991 ▸; Leonardi & Bish, 2016 ▸). This limits the use of the PDF compared with other techniques for analysis of powder scattering data (Billinge & Levin, 2007 ▸). In addition, the separation of the SAS particle shape contribution from the intensity profile is non-trivial. Approximating the SAS particle shape contribution by truncation at the intensity minimum before any Bragg contribution is a popular choice and common practice when analysing experimental data (Cargill, 1971 ▸; Farrow & Billinge, 2009 ▸; Mullen & Levin, 2011 ▸). However, the tails of the Bragg peaks extend underneath the profile, causing significant errors. Although experimental data are treated the same way, different approximation errors result from contributions that are neglected by the DSE, such as the interparticle cross-correlation.

Olds *et al.* (2015 ▸) proposed computing an approximate shape function via convolution of the RDF for a discrete model of the particle shape with either a sinc or the shape function of a sphere. Such an approach is flexible enough in principle to describe arbitrary particle shapes. But the use of the RDF limits the application to small nanoparticles and results in significant approximation errors. Any numerical RDF only approximates a continuous region of space. Gaps between lattice sites are not accurately corrected by the convolution of the RDF with any other function. The particle shape itself is a polyhedron bounded by a fixed set of crystallographic planes. To achieve optimal computing performance, Olds *et al.* proposed using the same RDF to compute the shape function and for modelling the reduced experimental data. However, the RDF cannot be corrected for the lattice distortion across this model. In addition, the most frequent pair distance between nearest-neighbour lattice sites is recorded in the RDF with the largest relative error because a constant-interval histogram RDF is generally implemented in computer algorithms (Hall & Monot, 1991 ▸; Leonardi & Bish, 2016 ▸). Ultimately, in the work of Olds *et al.*, the FT of the approximated shape function anomalously diverges from the DSE solution of perfect face-centred cubic (f.c.c.) nanocrystals for momentum transfer *Q* > 0.6 Å^−1^ despite the fact that the broadening of the nearest 220 peak and other reflections remains negligible for larger momentum transfers (*e.g.* *Q* 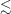 1 Å^−1^).

Numerical methods were used to tune an analytical model of the shape function using a training set (Usher *et al.*, 2018 ▸). The coefficients of overlapping Gaussian functions or *n*th-order polynomials were optimized against profiles computed across a range of particle sizes. Besides the possibility to extrapolate the solution to large nanoparticles, such a model improves accuracy by taking advantage of size-rescaling relations. However, the numerical method requires the calculation of many auxiliary RDFs. This renders tuning analytical models inefficient and generally infeasible for modelling powder scattering data.

Glatter, first, and Svergun more recently proposed using a linear combination of orthogonal functions to extract the shape function via modelling of the SAS particle shape contribution by direct FT (Svergun & Koch, 2003 ▸; Glatter, 1977 ▸). Although this is a more convenient and reliable approach than the inverse FT, the arbitrary choice of the functions inevitably affects the accuracy of the derived model. Developing upon this idea, here we propose to invert the order of the operations: first solving the spherical average and then the FT of the particle volume involved in the calculation of the form factors (Senesi & Lee, 2015*b* ▸; Scardi & Leoni, 2001 ▸; Leonardi *et al.*, 2012 ▸). The FT of the particle volume and the inverse FT of the SAS particle shape contribution then cancel each other. This allows FT operations to be disregarded, thus avoiding unnecessary calculations and approximations.

In this work, we compute the shape function from the directional components known as the common volume functions (CVFs). Closed-form expressions of the CVFs are known only for a few shapes, namely sphere, hollow sphere, cube, tetrahedron, octahedron, cylinder and hexagonal prism (Stokes & Wilson, 1942 ▸; Leoni, 2019 ▸; Lele & Anantharaman, 1966 ▸; Vargas *et al.*, 1983 ▸; Langford & Louër, 1982 ▸; Burresi & Tapfer, 2019 ▸). Here, we also report the closed-form expression of the CVFs for hollow cubes. Furthermore, discrete CVFs are readily available for many polyhedra from tables or can be calculated with numerical algorithms for any polyhedron of interest with arbitrary accuracy (Leonardi *et al.*, 2012 ▸; Leonardi, 2021 ▸). Compared with other methods, the use of CVFs improves accuracy of the shape function solution for arbitrary shapes and does not require the additional computation of any RDF or SAS particle shape contribution. We assess the accuracy of the shape function solution by modelling the SAS particle shape contribution to powder intensity profiles. We demonstrate that the computed shape functions accurately describe particle shape beyond resolution or size limitations. Finally, we investigate how particle size and dispersity affect the shape function.

## 2. Methods

### 2.1. Estimation of the shape function

The CVF describes the volume of the intersection of a shape with a copy of itself translated by a distance *r* along the direction 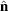 (Scardi & Leoni, 2001 ▸; Leonardi *et al.*, 2012 ▸). Given the shape 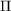 of a particle, the CVF 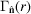 is proportional to the probability of a pair of sites separated by the vector 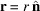 lying within 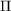 (Leonardi, 2021 ▸),

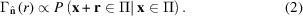

The shape function is then the orientational average of the CVF over Ω = 4π st,

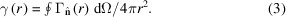

**Figure 1:**
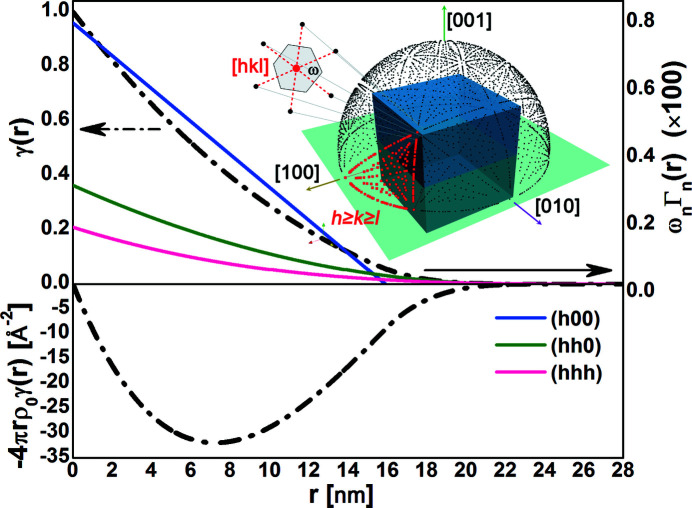
Modelling the shape function for a cube of edge length ∼16 nm. The shape function, 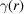 , is computed by summing the CVFs, 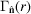 , over a set of 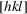 directions. Here the CVFs for the [100], [110] and [111] directions are shown as an example. The contribution for each direction was scaled with the area fraction, 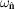 , of a unit-surface-area sphere divided by a tessellation for which the projections of the directions are generator centres (schematic inset). The small-angle PDF correction in equation (6) was then computed assuming 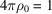 , *i.e.* by ignoring material composition.

Due to the nonlinear and discontinuous dependence of the CVF on the observation direction 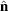 , the analytic solution of the integral is typically not possible. We compute a numerical approximation using a weighted average of the CVFs for a discrete set of directions 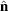 as (Fig. 1 ▸)

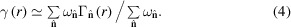

The directions in these sums are either read from tables or chosen as evenly distributed over the surface of the unit sphere. If the set of directions is read from tables, their projections onto the unit sphere are used as the nodes of a triangulation. The weight factors 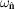 are calculated as the surface areas of the polygons that connect the barycentres of the triangular cells that share the direction projection as one of the cell’s corners. Although we use a Delaunay triangulation, the pattern of the direction projections can be adapted to best capture the particle shape with a finite set of directions. In contrast, if the set of directions is not fixed, they are sampled from a hemisphere { 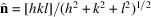 with 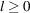 } at constant polar angle intervals with the zenith axis 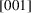 . For each polar angle, a subset of directions is sampled at constant azimuthal angle intervals. The weight factors 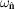 are calculated as the surface area of the portion of sphere surface in the angle range between two sequential polar and azimuthal angles. The number of azimuthal angle intervals 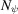 is then adjusted to yield an approximately constant area as

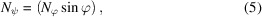

where 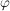 is the polar angle and 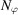 is the number of polar angle intervals. The point-group symmetry of a shape can be used to constrain the angular region to sample and further optimize the computing efficiency of the algorithm. As an example, we sample the azimuthal angles only in the first octant of the hemisphere because all shapes considered in this work, as well as most nanocrystals synthesized with controlled shape methods, have at least octahedral symmetry. If a finite number of CVFs is read from tables, an auxiliary triangulation is computed for the respective set of directions. The CVF for any direction with a projection lying within one of the auxiliary triangular cells is then approximated by the linear combination of the CVFs for the directions of the projections that bound that cell using the barycentric coordinates as weights (Leonardi, 2021 ▸).

### 2.2. Modelling powder scattering data

We model powder scattering data in real space and reciprocal space, the PDF and the intensity profile, respectively, with the whole pair distribution function modelling (WPDFM) method (Leonardi, 2021 ▸). Although the WPDFM does not use an atomistic model of a particle, we demonstrate the reliability of the modelled powder intensity profiles against profiles simulated via full solution of the DSE. Atomistic models of Pd single-crystal f.c.c. (unit cell 3.8907 Å) particles are built by selecting from an ideal lattice all sites enclosed in the particle shape. A set of 1000 equivalent models per shape are built by randomly shifting the particle relative to the lattice. The intensity profiles for these particle models are calculated with the software *Rose-X* (Debye, 1915 ▸; Leonardi & Bish, 2016 ▸), and their average is used to simulate the statistics of particles in the powder. This provides a match with the assumptions in the WPDFM method for the probability of observing a given atom pair distance in the volume of the particle shape. Given that the WPDFM estimates the RDF by exploiting the CVF, the scattering profiles and the shape function computed using equation (4) share the same accuracy and statistical approximations. The reduced PDF 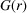 is then computed as

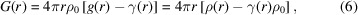

where 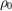 is the average atom density and 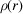 the pair density function (Egami & Billinge, 2003 ▸). Notably, in equation (6), the PDF 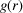 is corrected by the inverse of the shape function, which is a constant unit for bulk-like materials.

### 2.3. Modelling the small-angle contribution to intensity profiles

The intensity scattered by a powder of identical isolated crystals is

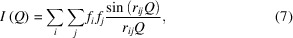

where 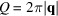 with the momentum transfer vector 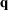 . *f* is the atomic scattering factor and 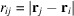 the pair distance between atoms *i* and *j*. Equation (7) is the most common formulation of the DSE. It can be rewritten as

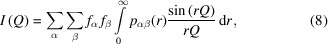

where 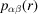 is the differential RDF for the elemental species 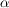 and 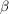 . Because our interest lies in the particle shape contribution, we neglect the atomic scattering factor contribution from now on and consider only the autocorrelation contribution to the intensity profiles. We assume monoatomic materials and set 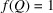 . The SAS particle shape contribution is then

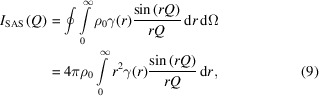

where the shape function is integrated over the sphere due to the powder assumption. We solve equation (9) for the numerical approximation of the shape function as

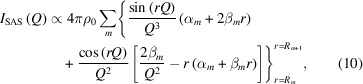

where 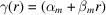 for *r* in the *m*th discrete set of translation distances [*i.e.* 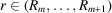 ] evaluated with equation (4).

## 3. Results and discussion

### 3.1. Shape function solution accuracy

Errors in the calculation of the intensity profile are magnified due to the duality between reciprocal and real space. Therefore, we validate the numerical approximation of the shape function by testing the agreement between the SAS particle shape contribution to the intensity profile computed with the shape function solving equation (10) and as present for small values of momentum transfer (*e.g.* 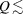 1 Å^−1^) in the powder intensity profile computed via DSE. We first probe the accuracy of our algorithm by exploiting the symmetry of a spherical shape for which 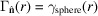 for all 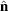 . For a sphere, the ideal solution of equation (9) is expressed by the Rayleigh formula (Rayleigh, 1910 ▸; Guinier & Fournet, 1955 ▸), which computes the SAS particle shape contribution normalized by the volume of the sphere (*i.e.* 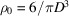 ) as

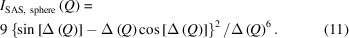

**Figure 2:**
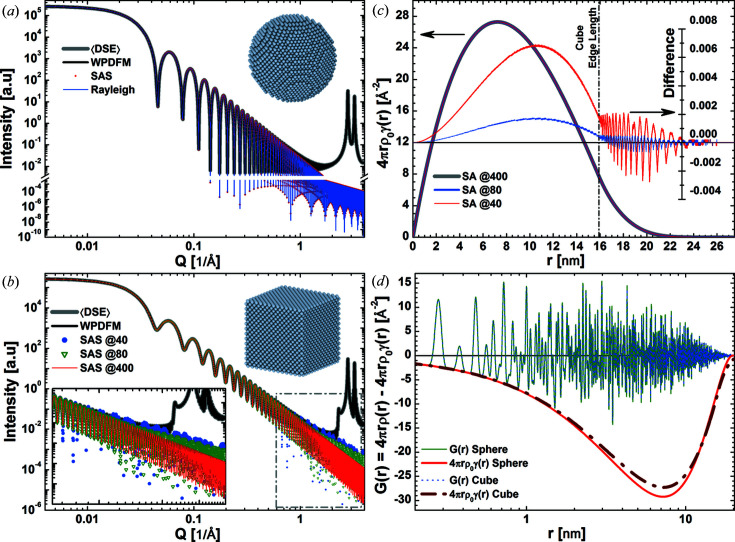
Accuracy of the SAS and shape function. Powder intensity profiles for a 20 nm spherical nanoparticle (*a*) and a 16 nm cubic nanoparticle (*b*) with f.c.c. (unit cell 3.8907 Å) structure and identical volumes. We compare the SAS particle shape contributions computed using the shape function with powder intensity profiles simulated with the DSE and the WPDFM method. The Rayleigh solution for the spherical particle is shown as a reference. (*b*) The SAS particle shape contribution for the cubic particle was computed using an increasing number of directions [*i.e.* (@40) 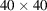 , (@80) 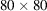 , (@400) 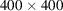 ] to approximate the shape function. (*c*) Shape function for the cubic particle computed using an increasing number of directions (left scale), and their difference (right scale) compared with the solution using the highest number of directions. (*d*) Reduced PDFs 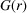 and negative shape function 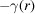 , which is the correction term in equation (6).

Here, 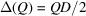 and *D* is the sphere diameter. The Rayleigh formula and the solution of equation (10) are in perfect agreement [Fig. 2 ▸(*a*)]. They overlap with the powder intensity profile in the limit of small momentum transfer, 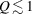 Å^−1^. For larger momentum transfer, 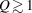 Å^−1^, the powder intensity profile grows because of the contribution from Bragg peak tails while the SAS particle shape contribution extends further down. We use a similar comparison to evaluate the reliability of the SAS particle shape contribution for other shapes that lack closed-form solutions.

We investigate the accuracy of the numerical approximation of the shape function for a cube to assess the reliability of our approach when using tabulated CVFs. The SAS particle shape contribution is calculated using either the closed-form expression of the CVFs or the discrete approximation from available tables. In particular, we use the discrete CVFs tabulated by Leonardi (2021 ▸) with a set of 163 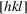 independent directions and 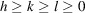 (Leonardi, 2021 ▸). Errors in the intensity profiles are marginal. Approximations in the shape function yield visible, yet small, errors in the intensity profile only for large momentum transfer, 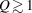 Å^−1^. The SAS particle shape contribution deviates from the expected trend because of the shape function error. The magnitude of noise errors and that of the deviation increase as a function of the momentum transfer and with the decrease of directional resolution of the tabulated CVFs. As a result of the summation in equation (4), these errors are inversely proportional to the number of sampled directions. In fact, the solid angle approximated by each sampled CVF increases with the decrease of directional resolution [Fig. 2 ▸(*b*)]. Each CVF approximates a larger set of directions along which the particle shape and its copy do not intersect for different limit translation distances. For pair distances larger than the cube edge length, the difference between shape functions computed with a different number of directions fluctuates with wavelength and amplitude inversely proportional to the directional resolution [Fig. 2 ▸(*c*)]. Such fluctuations result in a noisy error contribution to the intensity profile. Notably, this relation is reciprocal to that affecting the inverse FT of the SAS particle shape contribution. Although the shape function error may be significant in the intensity profile, the effect on the reduced PDF is usually negligible. Despite the dependence of the solution on the directional resolution for a cubic shape but not for a spherical shape, the reduced PDFs of f.c.c. nanocrystals with a different shape but identical volume perfectly overlap in the range of short pair distances [Fig. 2 ▸(*d*)]. This demonstrates that a small number of directions is enough to compute an accurate shape function suitable for correcting the PDF.

### 3.2. Shape function solution efficiency

**Figure 3:**
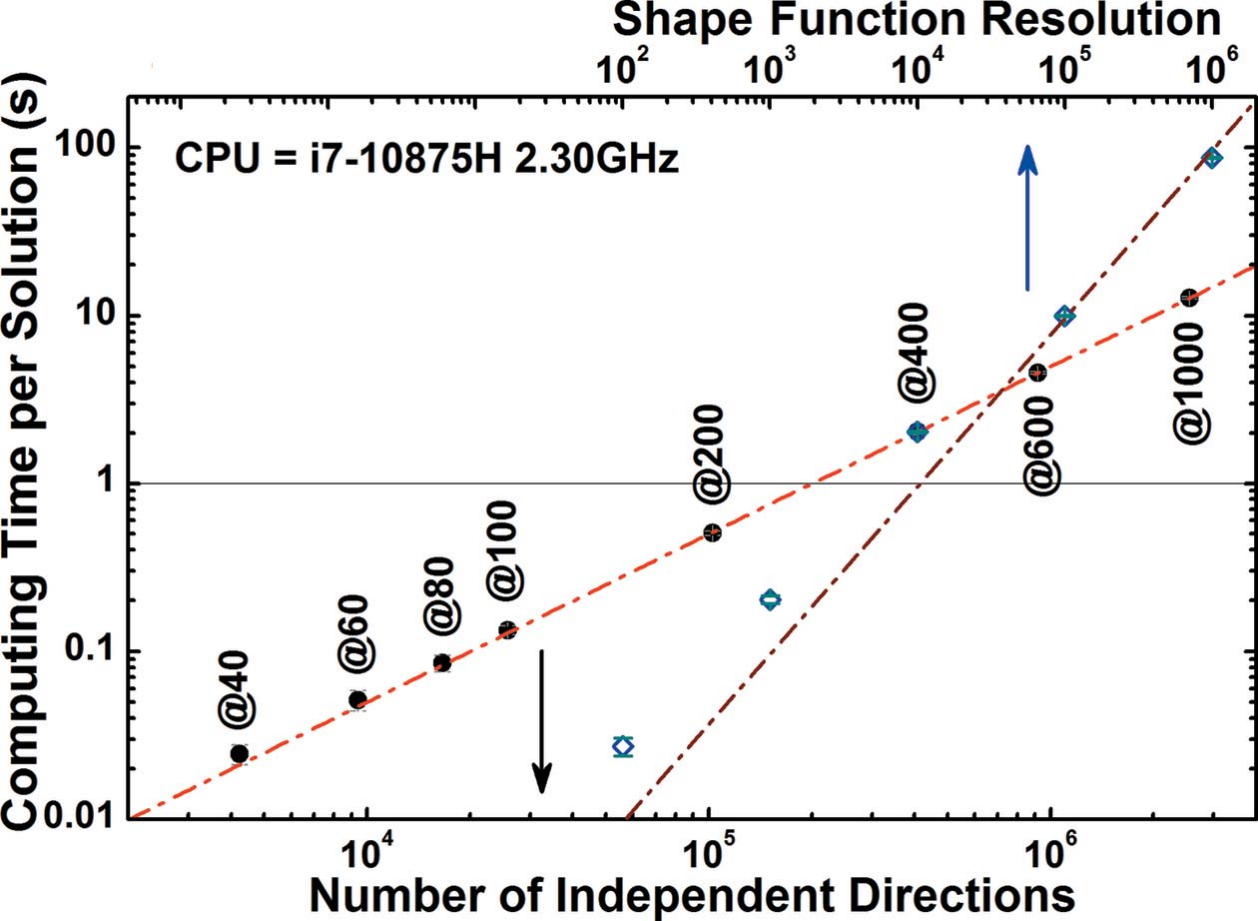
Analysis of computing performance. Time required to compute the shape function for a cube with (black full dots) a variable number of directions (or polar and azimuthal angle intervals, *e.g.* @40 for 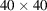 ) and 10 000 points per solution, or (blue open dot) 409 033 directions (*i.e.* @400) and a variable number of points per solution. The best-fit linear trends are also shown (red dash–dotted line). The deviation of the fit for the shape function resolution curve is caused by a constant initialization time.

We measure the performance of the algorithm implementation within a standalone C library as the time required to solve the shape function for a cubic particle using the closed-form expressions of the CVFs. A binary version of the code is available as supporting information. The size of the particle is irrelevant for performance because its effect is only a scaling factor. The computing time increases with the directional resolution and the number of points computed per shape function (Fig. 3 ▸). The relation deviates from linear for a small number of points calculated along the shape function. The apparent increase of computing time is explained by memory initialization. The computing time required to solve the shape function with sufficient directional resolution (16 609 or @80 directions) and number of points (5000) to either calculate an accurate intensity profile or correct a PDF is only 0.05 s. This allows real-time evaluation of the shape function within routines that optimize particle shape, size and their dispersion parameters.

### 3.3. Compact shapes

**Figure 4:**
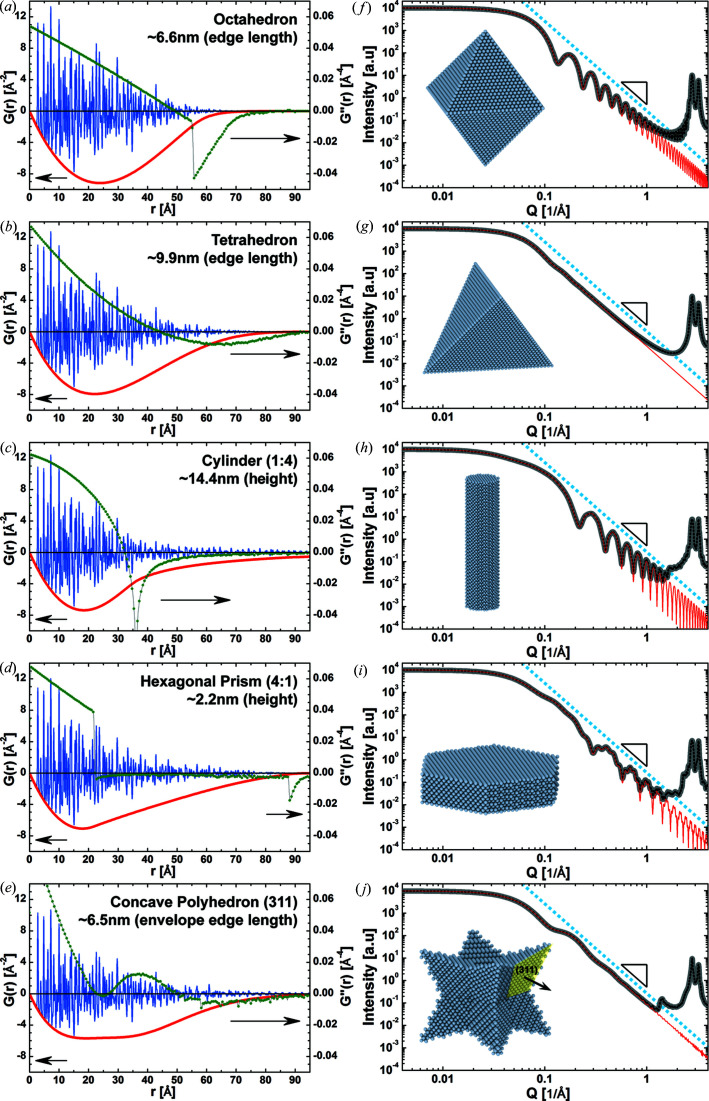
Reduced PDF *G*(*r*) and intensity scattering profiles from compact-shaped crystals. (*a*)–(*e*) Reduced PDF 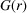 (blue), negative shape function 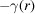 , which is the correction term in equation (6) (red), and (green) the second derivative of the negative shape function for powders of particles with octahedral (*a*), tetrahedral (*b*), cylindrical rod (*c*), plate-like hexagonal prism (*d*) and concave star (*e*) shapes. Sizes and size ratios are given in the figure legends. (*f*)–(*j*) Powder intensity profiles for the same shapes as in (*a*)–(*e*). SAS intensity profiles (red) are compared with the powder intensity profiles simulated with the DSE (grey) and the WPDFM method (black). The same black triangle is shown in each plot to mark the descending slope of the tail of the SAS contribution.

We compute the shape function and the SAS particle shape contribution to the intensity profile for various single-crystal f.c.c. nanoparticles (unit cell 3.8907 Å). In Fig. 4 ▸, the powder intensity profiles for an octahedron, a tetrahedron, a cylindrical rod, a plate-like hexagonal prism and a concave polyhedron are shown along with the reduced PDF and the negative shape function 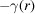 , which is the correcting term in equation (6). The accuracy of the solutions is supported by the facts that (i) the reduced PDFs oscillate about the pair distance axis and (ii) the SAS particle shape contributions to the intensity profiles completely overlap with the powder intensity profiles for small momentum transfer and then monotonically decrease under the Bragg peaks. The SAS particle shape contribution decreases with the same power (*i.e.* about −4.0) for all shapes because the particles were chosen with identical volume (*i.e.* about 140 nm^3^). Importantly, the solution for the concave polyhedron bounded by 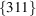 facets using CVF coefficients from numerical tables does not show any visible error up to large momentum transfer, 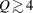 Å^−1^. This demonstrates the accuracy of our approach to compute the shape function for arbitrary shapes including concave and convex polyhedra, rods and star shapes.

The shape functions converge to zero for a pair distance either zero or equal to the diameter of the circumsphere of the crystal shape. They are right-skewed due to the convex CVF profiles of the compact shapes. A pronounced asymmetry appears for shapes with low sphericity. The less the particle shape resembles a sphere, the more diverse the translation distances at which the CVFs along different directions converge to zero. As an extreme case, flat and elongated particles (*e.g.* plate-like hexagonal prisms and cylindrical rods) are described by two distinct sets of CVFs depending on the shape, exposing a thin or thick projection along the direction of observation. Although the transition is ultimately continuous, the profile of the shape function splits into two regions with an elongated right tail.

Shape functions are continuous functions. They are usually smooth functions (Zygmund, 1945 ▸; Lorentz, 2010 ▸; Chen, 2010 ▸), but jumps or cusps appear in the second derivative profile for those particles bound by parallel atomic planes [Figs. 4 ▸(*a*), 4 ▸(*c*) and 4 ▸(*d*)]. Sites at the particle surface are then separated by a characteristic pair distance. These sites cause the pronounced ripples marking the SAS particle shape contribution to the intensity profiles (Li *et al.*, 2016 ▸). The frequency of the ripples is inversely proportional to the characteristic pair distance, whereas the amplitude is proportional to the integral surface of the site’s sets (*i.e.* the particle surface area). Different sets of sites with different characteristic pair distances and about the same integral surface area cause different harmonics. For example, although the octahedron and the cylindrical rod show a single frequency [Figs. 4 ▸(*f*) and 4 ▸(*h*)], several oscillations with different frequencies combine in the SAS particle shape contribution of the plate-like hexagonal prism [Fig. 4 ▸(*i*)].

### 3.4. Hollow shapes

**Figure 5:**
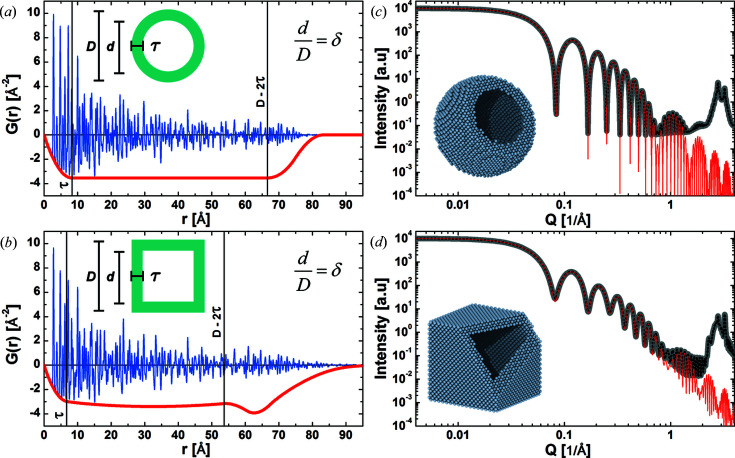
Reduced PDF *G*(*r*) and powder intensity profiles from hollow-shaped crystals. (*a*), (*b*) Reduced PDF 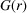 and negative shape function 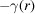 , which is the correction term in equation (6), for powders of particles with hollow spherical (*a*) and hollow cubic (*b*) shapes. The corresponding powder intensity profiles are shown in (*c*) and (*d*). SAS particle shape contributions to the intensity profiles (red) are compared with powder intensity profiles simulated with the DSE (grey) and the WPDFM (black) method.

Particle shapes with concentric hollow regions and uniform wall thickness represent a special class of morphology (Fig. 5 ▸). The uniform thickness of the walls results in a low-frequency modulation of the SAS particle shape contribution. The oscillation is visible against the background diffuse scattering of the powder intensity profile. We describe the closed-form expression of the CVFs for hollow sphere and hollow cube shapes by the sum of the CVFs for the envelope ( 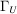 ) and the hollow region ( 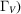 minus two times their intersection ( 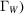 . These CVF terms are expressed with a piecewise polynomial as

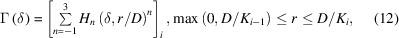

where the size ratio between the particle envelope and the hollow region is 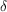 and the 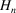 coefficients are listed in Tables 1 ▸ and 2 ▸ for the hollow sphere and hollow cube, respectively.

The CVFs of non-compact and non-convex shapes need not be monotonic. Although the volume of the intersection of a shape with its copy is positive and ultimately converges to zero, it can either decrease, remain steady or even increase with distance. This behaviour directly affects both the shape function and the SAS particle shape contribution to the intensity profiles. Compared with compact convex particle shapes, shape functions of hollow particles show a flat plateau in the range of pair distances between the wall thickness and the particle diameter minus two times the wall thickness, 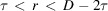 . Within this range, the CVF curves show the largest variation, whereas outside of this range they are only weakly affected by the hollowed morphology.

### 3.5. Particle dispersity

**Figure 6:**
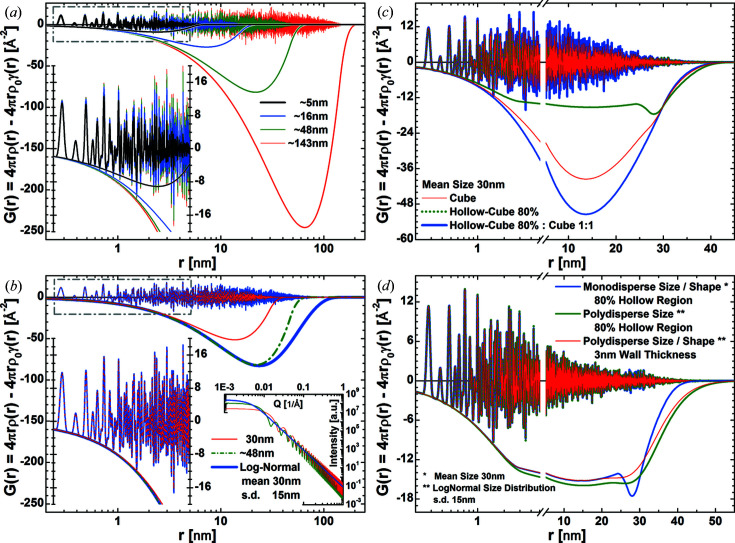
SAS contribution from size, and size and shape dispersion of crystals. Reduced PDFs 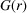 of crystals with f.c.c. (unit cell 3.8907 Å) structure and negative shape function 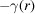 , which is the correction term in equation (6), for (*a*) monodisperse powders of cubic crystals with size ranging from ∼5 to ∼143 nm, (*b*) monodisperse and polydisperse powders of cubic crystals, (*c*) monodisperse powders of cubic, hollow cubic and a 1:1 mixture of these crystals, and (*d*) monodisperse powders of hollow cubic crystals and polydisperse powders of hollow cubic crystals. The short-range pair distances are magnified in the inset. The particle sizes of the monodisperse powders were chosen as the mean of the polydisperse system and the size yielding the best match of the shape functions. The left-side insets in (*a*) and (*b*) magnify the short-range pair distances. The right-side inset of (*b*) shows the SAS particle shape contributions to the intensity profile.

The effects of particle size and size dispersity in modelled powder scattering data are captured via scaling and convolution of the CVF, respectively. Both closed-form and tabulated CVFs are commonly normalized by the shape volume and a characteristic size parameter, *D*, such as the diameter for a sphere or the side edge length for a cube [see Table 1 of Scardi & Leoni (2001 ▸)]. The normalized CVF zeroes at a translation distance 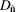 that is directly proportional to *D* [*i.e.* 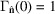 and 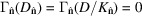 ] (Scardi & Leoni, 2001 ▸; Leonardi *et al.*, 2012 ▸). The proportionality factor 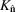 is a function of the direction 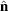 . Although the directional dependence is lost, a similar size proportionality still applies to the shape function. For example, the shape function shown in Fig. 6 ▸(*a*) for a sample of cubic particles scales exactly with particle size. Both the pair distances and their probability scale linearly with *D* [Fig. 6 ▸(*a*)]. The first scaling is due to the change in pair distance lengths that can be observed in the particle; the second scaling results from the pair distance scale factor in equation (6). Scaling the shape function affects the resolution (number of points per unit distance) and the accuracy of the calculated discrete function, making this route not a reliable option for accounting for different particle sizes. The evaluation of an accurate solution with a given resolution requires scaling the CVFs according to the target size and then solving equation (4). The broadening of the SAS particle shape contribution decreases with increasing particle size, pushing approximation errors towards smaller momentum transfer. Therefore, to achieve a given accuracy, the number of directions considered in the calculation of the shape function has to be adapted to particle shape and size. The error is inversely proportional to the smoothness of the particle surface and proportional to the misorientation angle between neighbouring facets. Although the behaviour outlined for the cubic shape provides a suitable reference, iterative algorithms can be used to adapt the number of directions explored achieving convergence. While the CVF may be known for a finite set of directions, interpolation methods have been proven reliable to capture the complete contribution (Leonardi, 2021 ▸).

Particle dispersity can be described by summing the shape functions of each shape and size in equation (4) as

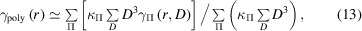

where each component is rescaled by the volume 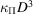 of the particle. For a chosen particle shape 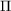 and a given size probability distribution 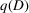 , equation (13) becomes

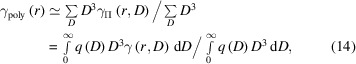

where the normalization integral is the third moment of the size probability distribution and the integral in the numerator is the convolution of the probability distribution with the shape function. Unless the shape function is known as a closed-form expression (*e.g.* for the spherical shape), its discrete approximation significantly affects the accuracy of the solution. To ensure high accuracy, the order of the integrations in equation (14) and equation (4) can be switched as

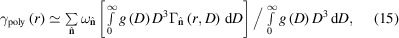

where the convolution in the numerator is now the same as that employed by the whole powder pattern modelling and WPDFM methods (Scardi & Leoni, 2001 ▸; Leonardi, 2021 ▸). Because CVFs are usually described by a third-order polynomial, the analytical solution is known for most common size probability distributions such as log-normal and gamma. Besides employing an analytical convolution, we calculate the shape function via numerical integration to prove the generality of the approach. Similar to any existing line profile analysis method, the computing performance and reliability of the solution are dependent on the degree of discretization. The larger the set of different sizes considered, the more partial solutions must be computed and therefore the lower the performance. Nonetheless, the finer the discretization, the better the modelled distribution can capture the size distribution in a real sample (Leoni & Scardi, 2004 ▸).

The size dispersity contribution is most significant for large distances in the PDF. The larger particles in a polydisperse system set the appearance and frequency of the longer pair distances. The deviation between the shape functions for mono- and polydisperse powders with the same mean particle size increases with increasing size dispersity and pair distance length. For example, a log-normal distribution of particles with standard deviation half the mean size yields a deviation 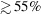 for a pair distance 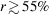 of the mean particle size [Fig. 6 ▸(*b*)]. A significantly larger size than the mean is required to adequately approximate the shape function of the polydisperse system. This demonstrates the danger of ignoring size dispersity effects in the analysis of PDF data. Shape dispersion yields similar artefacts. Although usually ignored, shape often changes with particle size. As an example, we consider the case of hollowed nanocrystals synthesized via galvanic replacement. The constant wall thickness yields a different size ratio, 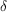 , between the particle envelope and the hollow region. A wall thickness of 3 nm for nanocrystals with envelope size from 40 to 12 nm results in a size ratio ranging from 85 to 50% [Figs. 6 ▸(*c*) and 6 ▸(*d*)].

## 4. Conclusions

We have illustrated that the shape function and the SAS particle shape contribution for a sample of nanoparticles with arbitrary shape and size can be efficiently calculated with high accuracy using CVFs. In contrast to other methods, for example the orientational average of the form factor or the trend of a whole-particle RDF (Senesi & Lee, 2015*b* ▸; Olds *et al.*, 2015 ▸; Wuttke, 2021 ▸), fits, interpolations or approximation parameters are not required. If the closed-form expressions of the CVFs are unknown, the CVF evaluated at a discrete set of translation distances and directions can be used instead. The resolution of the discretization was tuned to optimize accurate modelling of SAS particle shape contributions over a wide range of momentum transfer. The calculated scattering profiles converged to the exact solution with increasing particle shape resolution in the model description. In contrast to the approach proposed by Olds *et al.* (2015 ▸), the calculation of an auxiliary RDF was not required. Our approach is well suited for integration into numerical analysis tools of powder total scattering data that do not need the whole RDF. It also enables the consistent modelling of the whole powder scattering profile. Indeed, the same model information, the CVFs, can be used to model both the small-angle and the Bragg components, for example, via whole powder pattern modelling and PDF modelling methods (Scardi & Leoni, 2001 ▸; Leonardi, 2021 ▸).

We demonstrated that the size probability distribution of the polydisperse powder samples can be convoluted with the shape function. The contribution from size dispersion was accounted for at a small additional computing cost compared with the solution for a monodisperse powder. Accurate shape functions and SAS particle shape contributions to the intensity profiles were computed efficiently for powders of large nanoparticles as well as for large size dispersion. Our approach overcomes limitations of direct methods, that are particularly severe under those conditions (Neder & Proffen, 2020 ▸).

We reviewed how particle shape affects the SAS contribution to intensity profiles. CVFs are typically known in closed form or via tabulated coefficients for a wide scenario of particle shapes. In addition, we derived the analytical CVF expressions for hollowed spheres and hollowed cubes. While sinusoids in the SAS particle shape contribution can appear as a result of the truncation of the coefficient series in the FT, we showed that the source of ripples is encoded in the second derivative of the shape function. Distinct behaviours were observed in the shape function for concave and hollowed shapes. Accounting for deformations of known particle shapes is left for future work.

## Supplementary Material

Binary software. DOI: 10.1107/S1600576722001261/jl5028sup1.zip

## Figures and Tables

**Table 1:** Coefficients for the analytical expression of the CVF for a hollow sphere Note that 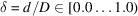 .

	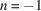	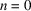	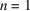	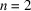	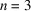	*K*
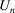	0	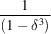	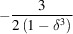	0	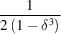	1
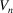	0	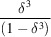	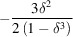	0	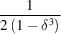	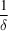
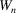	0	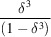	0	0	0	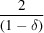
	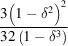	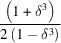	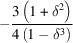	0	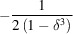	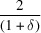

**Table 2:** Coefficients for the analytical expression of the CVF for a hollow cube We index the directions using positive indices with 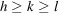 . This assumption does not limit the generality of the equations because of the cubic symmetry of the shape. Then 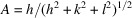 , 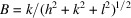 and 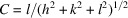 . Note that 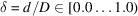 .

	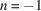	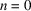	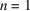	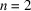	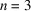	*K*
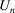	0	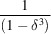	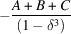	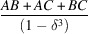	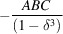	*A*
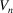	0	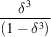	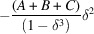	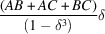	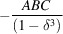	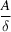
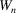	0	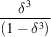	0	0	0	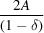
	0	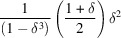	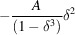	0	0	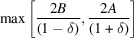
	0	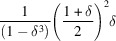	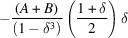	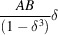	0	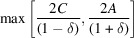
	0	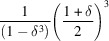	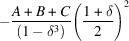	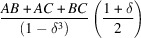	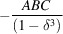	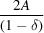

## References

[bb1] Azaroff, L. V. (1968). *Elements of X-ray Crystallography*. New York: McGraw-Hill Companies.

[bb2] Bartlett, P. & Ottewill, R. H. (1992). *J. Chem. Phys.* **96**, 3306–3318.

[bb3] Billinge, S. J. L. & Egami, T. (1993). *Phys. Rev. B*, **47**, 14386–14406. 10.1103/physrevb.47.14386 10005789

[bb4] Billinge, S. J. L. & Levin, I. (2007). *Science*, **316**, 561–565. 10.1126/science.1135080 17463280

[bb5] Burresi, E. & Tapfer, L. (2019). *Nanomater. Nanotechnol.* **9**, 184798041983238.

[bb6] Cargill, G. S. (1971). *J. Appl. Cryst.* **4**, 277–283.

[bb7] Chen, L. (2010). *arXiv*:1005.3727.

[bb8] Chen, Z., Zhang, X. & Lu, G. (2017). *J. Phys. Chem. C*, **121**, 1964–1973.

[bb9] Debye, P. (1915). *Nachr. Ges. Wiss. Göttingen*, **27**, 70–76.

[bb10] Egami, T. & Billinge, S. J. L. (2003). *Underneath the Bragg Peaks: Structural Analysis of Complex Materials*. Cambridge: Elsevier.

[bb11] Farrow, C. L. & Billinge, S. J. L. (2009). *Acta Cryst.* A**65**, 232–239. 10.1107/S0108767309009714 19349667

[bb12] Farrow, C. L., Juhas, P., Liu, J. W., Bryndin, D., Božin, E. S., Bloch, J., Proffen, T. & Billinge, S. J. L. (2007). *J. Phys. Condens. Matter*, **19**, 335219. 10.1088/0953-8984/19/33/335219 21694142

[bb13] Gamler, J. T. L., Leonardi, A., Sang, X., Koczkur, K. M., Unocic, R. R., Engel, M. & Skrabalak, S. E. (2020). *Nanoscale Adv.* **2**, 1105–1114. 10.1039/d0na00061b PMC9419249 36133036

[bb14] Glatter, O. (1977). *J. Appl. Cryst.* **10**, 415–421.

[bb15] Gong, M., Jin, X., Sakidja, R. & Ren, S. (2015). *Nano Lett.* **15**, 8347–8353. 10.1021/acs.nanolett.5b04036 26536534

[bb16] Goodisman, J. (1980). *J. Appl. Cryst.* **13**, 132–134.

[bb17] Guinier, A. (1956). *X-ray Diffraction*. Paris: Dunod.

[bb18] Guinier, A. & Fournet, G. (1955). *Small-Angle Scattering of X-rays*. New York: John Wiley and Sons.

[bb19] Hajfathalian, M., Gilroy, K. D., Golze, S. D., Yaghoubzade, A., Menumerov, E., Hughes, R. A. & Neretina, S. (2016). *ACS Nano*, **10**, 6354–6362. 10.1021/acsnano.6b02712 27172588

[bb20] Hall, B. D. & Monot, R. (1991). *Comput. Phys.* **5**, 414.

[bb21] Howell, R. C., Proffen, T. & Conradson, S. D. (2006). *Phys. Rev. B*, **73**, 094107.

[bb22] Hua, X., Liu, Z., Bruce, P. G. & Grey, C. P. (2015). *J. Am. Chem. Soc.* **137**, 13612–13623. 10.1021/jacs.5b08434 26422761

[bb23] Kodama, K., Iikubo, S., Taguchi, T. & Shamoto, S. (2006). *Acta Cryst.* A**62**, 444–453. 10.1107/S0108767306034635 17057353

[bb25] Korsunskiy, V. I. & Neder, R. B. (2005). *J. Appl. Cryst.* **38**, 1020–1027.

[bb24] Korsunskiy, V. I., Neder, R. B., Hofmann, A., Dembski, S., Graf, C. & Rühl, E. (2007). *J. Appl. Cryst.* **40**, 975–985.

[bb26] Langford, J. I. & Louër, D. (1982). *J. Appl. Cryst.* **15**, 20–26.

[bb27] Lele, S. & Anantharaman, T. R. (1966). *Proc. Indian Acad. Sci.* **64**, 261–274.

[bb28] Leonardi, A. (2021). *IUCrJ*, **8**, 257–269. 10.1107/S2052252521000324 PMC7924235 33708402

[bb29] Leonardi, A. & Bish, D. L. (2016). *J. Appl. Cryst.* **49**, 1593–1608.

[bb30] Leonardi, A. & Engel, M. (2018). *ACS Nano*, **12**, 9186–9195. 10.1021/acsnano.8b03759 30075066

[bb31] Leonardi, A., Leoni, M., Siboni, S. & Scardi, P. (2012). *J. Appl. Cryst.* **45**, 1162–1172.

[bb32] Leoni, M. (2019). *International Tables for Crystallography*, Vol. H, *Powder Diffraction*, 1st online ed., pp. 524–537. Chester: International Union of Crystallography.

[bb33] Leoni, M. & Scardi, P. (2004). *J. Appl. Cryst.* **37**, 629–634.

[bb34] Li, T., Senesi, A. J. & Lee, B. (2016). *Chem. Rev.* **116**, 11128–11180. 10.1021/acs.chemrev.5b00690 27054962

[bb35] Li, X., Shew, C.-Y., He, L., Meilleur, F., Myles, D. A. A., Liu, E., Zhang, Y., Smith, G. S., Herwig, K. W., Pynn, R. & Chen, W.-R. (2011). *J. Appl. Cryst.* **44**, 545–557.

[bb36] Lorentz, G. G. (2010). *J. Approximation Theory*, **75**, 1–7.

[bb37] Luo, M. & Guo, S. (2017). *Nat. Rev. Mater.* **2**, 17059.

[bb38] Mullen, K. & Levin, I. (2011). *J. Appl. Cryst.* **44**, 788–797.

[bb39] Neder, R. B. & Korsunskiy, V. I. (2005). *J. Phys. Condens. Matter*, **17**, S125–S134.

[bb40] Neder, R. B. & Proffen, Th. (2020). *J. Appl. Cryst.* **53**, 710–721. 10.1107/S1600576720004616 PMC7312130 32684886

[bb41] Olds, D., Wang, H.-W. & Page, K. (2015). *J. Appl. Cryst.* **48**, 1651–1659.

[bb42] Page, K., Hood, T. C., Proffen, Th. & Neder, R. B. (2011). *J. Appl. Cryst.* **44**, 327–336.

[bb43] Polking, M. J., Han, M.-G., Yourdkhani, A., Petkov, V., Kisielowski, C. F., Volkov, V. V., Zhu, Y., Caruntu, G., Paul Alivisatos, A. & Ramesh, R. (2012). *Nat. Mater.* **11**, 700–709. 10.1038/nmat3371 22772655

[bb44] Pospelov, G., Van Herck, W., Burle, J., Carmona Loaiza, J. M., Durniak, C., Fisher, J. M., Ganeva, M., Yurov, D. & Wuttke, J. (2020). *J. Appl. Cryst.* **53**, 262–276. 10.1107/S1600576719016789 PMC6998781 32047414

[bb45] Rayleigh, Lord (1910). *Proc. R. Soc. London*, **84**, 25–46.

[bb46] Renaud, G., Lazzari, R. & Leroy, F. (2009). *Surf. Sci. Rep.* **64**, 255–380.

[bb47] Scardi, P., Leonardi, A., Gelisio, L., Suchomel, M. R., Sneed, B. T., Sheehan, M. K. & Tsung, C.-K. (2015). *Phys. Rev. B*, **91**, 155414.

[bb48] Scardi, P. & Leoni, M. (2001). *Acta Cryst.* A**57**, 604–613. 10.1107/s0108767301008881 11526309

[bb49] Scardi, P., Leoni, M. & Beyerlein, K. R. (2011). *Z. Kristallogr.* **226**, 924–933.

[bb51] Senesi, A. J. & Lee, B. (2015*a*). *J. Appl. Cryst.* **48**, 1172–1182.

[bb50] Senesi, A. & Lee, B. (2015*b*). *J. Appl. Cryst.* **48**, 565–577.

[bb52] Solla-Gullon, J., Garnier, E., Feliu, J. M., Leoni, M., Leonardi, A. & Scardi, P. (2015). *J. Appl. Cryst.* **48**, 1534–1542.

[bb53] Stokes, A. R. & Wilson, A. J. C. (1942). *Math. Proc. Camb. Philos. Soc.* **38**, 313–322.

[bb54] Svergun, D. I. & Koch, M. H. J. (2003). *Rep. Prog. Phys.* **66**, 1735–1782.

[bb55] Usher, T.-M., Olds, D., Liu, J. & Page, K. (2018). *Acta Cryst.* A**74**, 322–331. 10.1107/S2053273318004977 29978843

[bb56] Vargas, R., Louër, D. & Langford, J. I. (1983). *J. Appl. Cryst.* **16**, 512–518.

[bb57] Wang, H.-W., Wesolowski, D. J., Proffen, T. E., Vlcek, L., Wang, W., Allard, L. F., Kolesnikov, A. I., Feygenson, M., Anovitz, L. M. & Paul, R. L. (2013). *J. Am. Chem. Soc.* **135**, 6885–6895. 10.1021/ja312030e 23607732

[bb58] Wuttke, J. (2017). *arXiv*:1703.00255.

[bb59] Wuttke, J. (2021). *J. Appl. Cryst.* **54**, 580–587. 10.1107/S1600576721001710 PMC8056765 33953657

[bb60] Zygmund, A. (1945). *Duke Math. J.* **12**, 455–464.

